# Infiltrative tumor growth patterns on magnetic resonance imaging associated with systemic inflammation and oncological outcome in patients with high-grade soft-tissue sarcoma

**DOI:** 10.1371/journal.pone.0181787

**Published:** 2017-07-20

**Authors:** Tomoki Nakamura, Akihiko Matsumine, Takao Matsubara, Kunihiro Asanuma, Yuki Yada, Tomohito Hagi, Akihiro Sudo

**Affiliations:** Department of Orthopaedic Surgery, Mie University Graduate School of Medicine, Tsu-city, Mie, Japan; Universite de Nantes, FRANCE

## Abstract

**Objective:**

The aim of this study was to determine whether the tumor infiltrative growth pattern on magnetic resonance imaging (MRI) was associated with blood inflammatory markers (C-reactive protein; CRP and Neutrophil-lymphocyte ratio; NLR) and survival in patients with high-grade soft-tissue sarcoma (STS).

**Methods:**

The cohort for this retrospective study included 81 patients with a mean age of 63 years. The tumor depth was superficial or deep in 15 and 66 patients, respectively. The mean CRP and NLR were 1.31 mg/dL and 2.81, respectively. The assessment of a peripheral growth pattern which divided into three patterns on MRI was based on the largest midsection of the tumor.

**Results:**

On MRI scans, diffuse-type, focal-type, and pushing-type growth patterns were observed in 18, 33, and 30 patients, respectively. Superficial high-grade STS were prone to show a focal-type pattern on MRI. There were no correlations between growth pattern type and clinicopathological factors such as age, sex, tumor size, and histological grade. However, the incidence of infiltrative growth was significantly higher in patients with elevated CRP (p = 0.0002). In multivariate analysis, growth pattern and CRP were independent prognostic factors for disease-specific survival, metastasis-free survival. Growth pattern was also related to local tumor control.

**Conclusions:**

There were significant associations between the tumor growth pattern and CRP levels in patients with high-grade soft-tissue sarcoma. An infiltrative growth pattern and elevated CRP may be associated with inferior disease-specific and metastasis-free survival rates in these patients. Therefore, careful post-treatment follow-up should be conducted in such patients.

## Introduction

In patients with soft-tissue sarcoma, the presence of systemic inflammation has been associated with a poor prognosis [1–6]. Elevations in the inflammatory markers, C-reactive protein (CRP) and the neutrophil:lymphocyte ratio (NLR) have been correlated with advanced histological tumor grade and size [1–6]. Therefore, the existence of systemic inflammation is indicative of aggressive tumor characteristics.

On the other hand, local tumor growth patterns can affect the clinical outcome in patients with soft-tissue sarcoma. In particular, the infiltrative growth pattern on magnetic resonance imaging (MRI) has been evaluated in both undifferentiated pleomorphic sarcoma (UPS) and myxofibrosarcoma (MFS) [7–12]. Infiltrative growth has been significantly correlated with poor disease-specific survival, metastasis-free survival, and local control, suggesting that it is a clinical indicator that reflects the aggressive behavior of tumors [7,9–12].

Because both systemic inflammation and infiltrative growth have been associated with poor prognosis and aggressive disease, we hypothesized that systemic inflammation might be related to the infiltrative MRI growth pattern. The aim of this study was to (a) determine whether there was a relationship between tumor border and inflammatory markers, (b) there was a relationship between tumor border and oncological outcome and (c) there was a relationship between inflammatory markers and oncological outcome.

## Patients and methods

Between August 2004 and October 2015, we treated 119 patients who had a histological diagnosis of a primary high-grade soft-tissue sarcoma. The patients who received unplanned excisions were excluded. Tumor grade was defined according to the French Federation of Cancer Centers Sarcoma Group system, and grade 2–3 disease was considered high-grade. Eight patients with lung metastasis at presentation were excluded from this study. Ten patients who did not undergo surgical tumor resection were also excluded. Two patients were excluded because of those with retroperitoneal tumor. Finally, 18 patients who had incomplete clinical histories or laboratory data were excluded. The final analysis cohort for this retrospective study included 81 patients. Written informed consent was obtained from each patient at the time of admission or treatment. The cohort included 50 males and 31 females with a mean age of 63 years. The mean follow-up duration was 45 months (range, 5–136 months). The primary tumor sites included the thigh (n = 29), lower leg (n = 13), buttock (n = 10), back (n = 3), chest wall (n = 6), upper arm (n = 6), and others (n = 14). The tumors were histologically classified as follows according to WHO classification: 20 UPSs, 15 leiomyosarcomas (LMSs), 15 MFSs, 13 liposarcomas (LPSs), 7 malignant peripheral nerve sheath tumors (MPNSTs), and 13 other tumors. Twenty-four and 59 patients had grade 2 and grade 3 sarcomas, respectively. The mean tumor size at diagnosis was 9.3 cm (range, 2–30 cm, Median 7.5cm). The tumor depth was superficial or deep in 15 and 66 patients, respectively. The mean CRP was 1.31 mg/dL (range, 0.01–20.4 mg/dL, Median, 0.2 mg/dL). The mean NLR was 2.81 (range, 0.91–9.34, Median 2.32).

Seventy-eight patients underwent surgical tumor resection and the remaining 3 patients underwent amputation. The surgical margin was not evaluated in 4 patients who underwent extracorporeal irradiation and autologous bone graft after tumor resection with bone involvement. Microscopic surgical margin status was positive in 12 patients and negative in 67 patients. Six patients were administered adjuvant radiotherapy, and 20 underwent chemotherapy.

Pretreatment workup included contrast-enhanced and non-contrast-enhanced computed tomography of the lung, abdomen, and pelvis. MRI and blood samples were obtained before treatment in all patients. The NLR was defined as the absolute neutrophil count divided by absolute lymphocyte count. This study was approved by institutional review of board at Mie University Hospital. Written informed consent was obtained from all patients. This study was conducted in accordance with the Declaration of Helsinki Principles.

### Assessment of MRI and definition of tumor growth pattern

**Fig 1:**
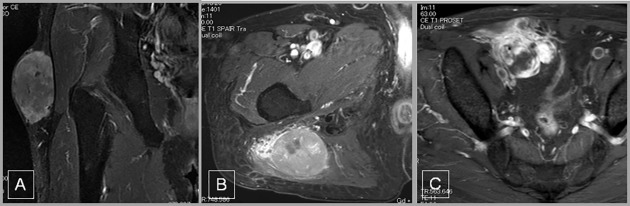
Infiltrative growth patterns on MRI. (a) pushing-type (b) focal-type and (c) diffuse-type.

All patients underwent preoperative imaging including T1-weighted MRI, T2-weighted MRI, short-TI Inversion Recovery (STIR) and Gadolimium (Gd)-MRI. STIR or Gd-MRI was evaluated for signal characteristics and a tumor-infiltrative growth pattern.^10^ The assessment of a peripheral growth pattern on MRI was based on the largest midsection of the tumor.^11^ “Pushing-type” was considered when the tumor was well defined without peripheral extension to the surrounding tissue, whereas a tumor was classified as infiltrative if it had an irregular surface and invaded the surrounding tissue. Infiltrative growth was classified as “focal-type (IF)” (<25% of the tumor circumference) or “diffuse-type (ID)” (>25% of the tumor circumference) (**Fig 1**). For the patients with an infiltrative tumor pattern on MRI, tumor size was measured at the greatest dimension, except for at portions showing an irregular surface and invasion into the surrounding tissue [9].

### Statistical analyses

Statistical associations between clinicopathological variables were evaluated using the Mann-Whitney U-test and the Kruskal-Wallis test for quantitative data, and the χ^2^ test for qualitative data. The survival time was measured from the date of the initial treatment for the primary tumor to the date of last follow-up or death due to sarcoma. The local recurrence and metastasis-free survival were measured from the date of the initial treatment for the primary tumor to the date of development of local recurrence or metastasis, respectively. We usually evaluate local site every 6 months using MRI findings and metastasis every 3 or 4 months using CT scan. Survival curves were constructed using the Kaplan-Meier method. Univariate and multivariate analyses were performed using log-rank test and Cox proportional hazard model. The variables included in the multivariate analysis were those identified as significant in the univariate analysis. The log-rank test was used to compare survival, recurrence, and metastasis. A value of p < 0.05 was considered significant for all statistical analyses. All analyses were conducted using the statistical software package Stat View Version 5.0 (SAS Institute, Cary, NC, USA).

## Results

### Growth pattern and clinical factors

On MRI scans, ID-type, IF-type, and pushing-type growth patterns were observed in18, 33, and 30 patients, respectively. There were no correlations between growth pattern type and clinicopathological factors such as age, sex, tumor size, and histological grade. There was also no relationship between surgical margin status and infiltrative growth pattern. Concerning superficial STS, 9 of 15 sarcomas consisted of UPS, MFS. Additionally, 8 of 9 patients with superficial UPS and MFS showed IF-type growth pattern. Histologically, IF-type pattern on MRI was often observed in patients with UPS, MFS and LMS, whereas ID-type in de-differentiated LPS and MPNST. Furthermore, the incidence of infiltrative growth (IF-type or ID type) was significantly higher in patients with elevated CRP (p = 0.002) (**Table 1**).

**Table 1:** The relationship between MRI growth pattern and clinical data.

	MRI pattern			p-value
Variables	pushing	focal	Diffuse	
Mean age (yrs)	63	62	64	0.99*
Sex				
Male	14	24	12	0.09
Female	16	9	6	
Grade				
G2	10	8	6	0.68
G3	20	25	12	
Mean size (cm)	8.6	9.1	11	0.72
Depth				
Superficial	4	10	1	0.06
Deep	26	23	17	
Histology				
UPS	6	11	2	0.001
MFS	6	6	3	
LMS	6	8	1	
LPS (de-differentiated type)	8 (4)	0	5 (4)	
MPNST	1	0	5	
Others	3	8	2	
Surgical margin				
Negative	23	29	13	0.13
Positive	5	2	5	
Median CRP levels (mg/dl)	0.11	0.5	0.55	0.002
Median NLR	2.15	2.27	3.43	0.05

CRP; C-reactive proteon, NLR; Neutrophil:lymphocyte ratio, UPS; Undifferentiated pleomorphic sarcoma, MFS; Myxofibrosarcoma, LMS; Leiomyosarcoma, LPS; Liposarocma, MPNST; Malignant peripheral nerve sheath tumor.

*The Kruskal-Wallis tests was used; for the rest of the results, the Chi square test was used.

### Prognostic factor analyses

At the final follow-up, 23 patients had died of the disease and 2 had died from other causes. The 3-year and 5-year disease-specific survival rate was 77.3% (95% confidence interval [CI], 67.7–86.9) and 66.7% (95%CI, 52.2–77.9), respectively.

**Fig 2:**
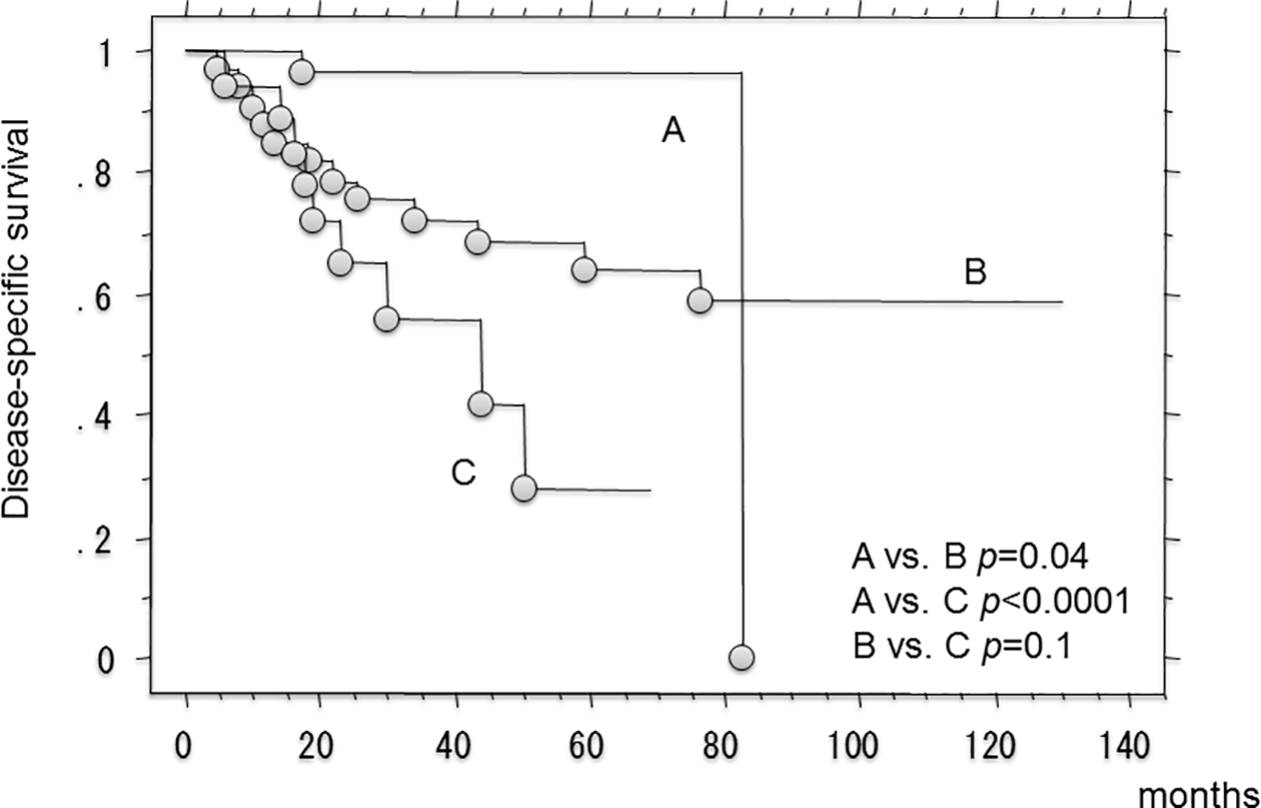
Kaplan-Meier curve showing the disease-specific survival rates in 81 patients with soft-tissue sarcomas. (A; patients with pushing-type infiltrative pattern, B; patients with focal-type infiltrative pattern, C; patients with diffuse-type infiltrative pattern).

Univariate analysis for disease-specific survival confirmed the predictive value of growth pattern (pushing vs. ID: p = 0.004, pushing vs. IF: p = 0.08), CRP (p <0.0001), and tumor size (p = 0.005). In the multivariate analysis, growth pattern (pushing vs.ID: p = 0.02), CRP (p = 0.002), and tumor size (p = 0.01) were independent prognosticators for disease-specific survival (**Table 2**). The 3- and 5-year disease-specific survival rates in patients with ID-type growth were 55.7% (95%CI, 29.8–81.6) and 27.9% (95% CI, 0–58.1), respectively, compared with 72.1% (95%CI, 56.6–87.6) and 63.8% (95%CI, 46.2–81.4) in those with IF-type growth (p = 0.04, log rank test). Those with ID-type growth also had inferior disease-specific survival compared to those with pushing-type growth (p < 0.0001, log rank test). The 3- and 5-year disease-specific survival rates in patients with pushing-type were 96.6% (95%CI, 89.9–100) and 96.6% (95%CI, 89.9–100), respectively (**Fig 2**). There was also significant differences in disease-specific survival rates between patients with IF-type and pushing-type growth (p = 0.04, log rank test). When we divided into 2 groups according to the median levels of CRP, NLR, size and infiltrative pattern (IF + ID vs. Pushing type), those were significantly related to survival (**Table 3**).

**Table 2:** The prognostic factors for disease-specific survival in 81 patients.

Variables		Univariate analysis			Multivariate analysis		
		HR	95% CI	p-value	HR	95% CI	p-value
Age	years	1.015	0.987–1.043	0.29			
Gender	female	1.534	0.677–3.481	0.31			
	male	1					
Grade	G2	0.675	0.25–1.827	0.44			
	G3	1					
Size	cm	1.093	1.027–1.162	0.005	1.086	1.019–1.15	0.01
Depth	deep	1.173	0.397–3.468	0.77			
	s/c	1					
CRP	mg/dl	1.165	1.087–1.249	<0.0001	1.126	1.046–1.21	0.002
NLR		1.257	0.983–1.608	0.07			
Growth	pushing	0.103	0.022–0.477	0.004	0.156	0.032–0.76	0.02
pattern	focal	0.437	0.174–1.1	0.08	0.51	0.194–1.34	0.17
	diffuse	1			1		

CRP; C-reactive protein, NLR; Neutrophil-lymphocyte ratio, HR; Hazard ratio, 95%CI; 95% confidential interval

**Table 3:** Univariate analysis of CRP, NLR and tumor size using log-rank test.

Variables	n	5Y-DSS	p value	5Y-LRFS	p value	5Y-MFS	p value
CRP< 1.4mg/dl	69	70.6%	0.01	80.2%	0.12	56.9%	0.047
CRP≥ 1.4mg/dl	12	38.9%		NR		41.7%	
NLR< 2.8	45	80.2%	0.01	81.7%	0.47	67.4%	0.07
NLR≥ 2.8	36	51.9%		70.0%		42.2%	
Size< 9cm	45	80.2%	0.04	87.9%	0.004	63.6%	0.36
Size≥ 9cm	36	47%		61.3%		45.7%	
IF+ID	51	54.5%	0.007	65.7%	0.03	38.6%	0.002
Pushing	30	96.6%		93.2%		89.9%	

CRP; C-reactive protein, NLR; Neutrophil-lymphocyte ratio, DSS; Disease-specific survival, LRFS; Local recurrence-free survival, MFS; Metastasis-free survival, IF; focal-type infiltrative growth pattern, ID; Diffuse-type infiltrative pattern

**Fig 3:**
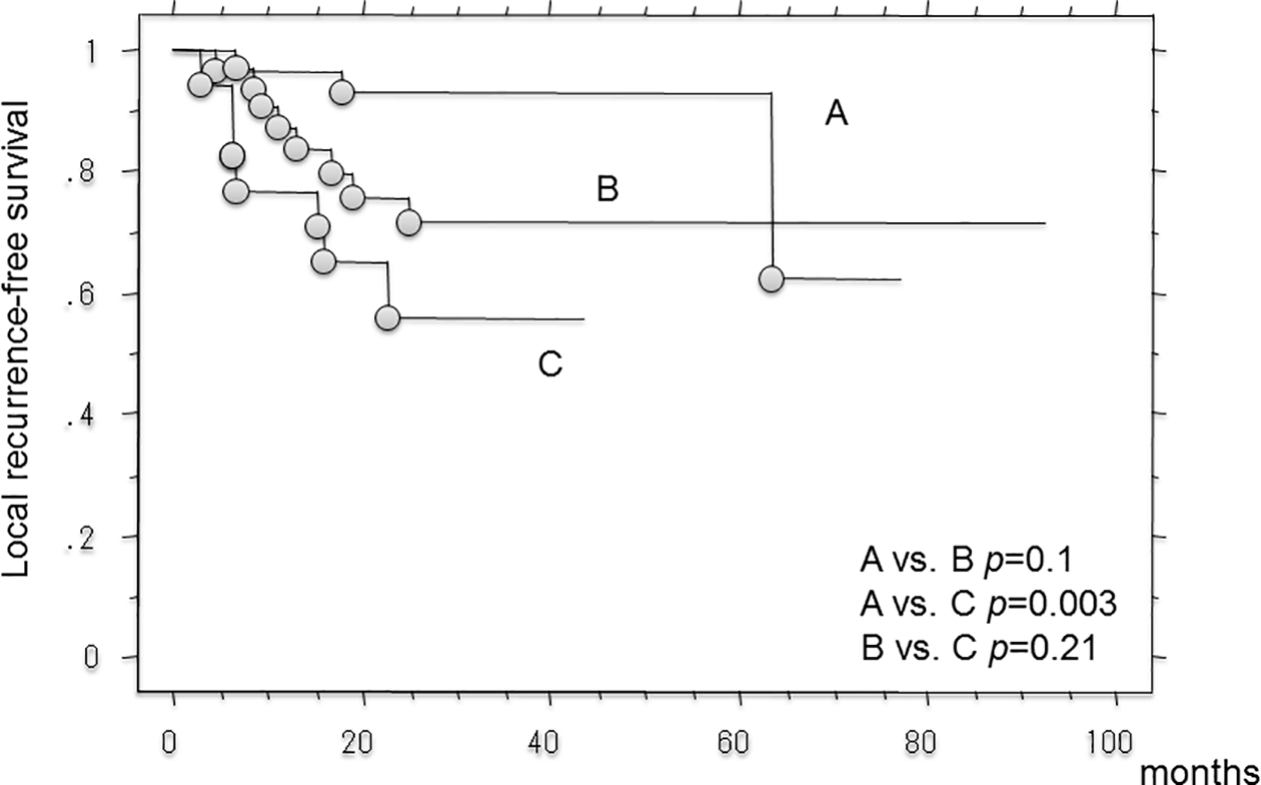
Kaplan-Meier curve showing the local recurrence-free survival in 81 patients with soft-tissue sarcomas. (A; patients with pushing-type infiltrative pattern, B; patients with focal-type infiltrative pattern, C; patients with diffuse-type infiltrative pattern).

Local recurrence was observed in 18 patients. The 3-year and 5-year local recurrence-free survival was both 76.7% (95%CI, 66.9–86.6). On univariate and Multivariate analysis, smaller size and pushing growth pattern (vs. ID type) had worse local recurrence-free survival (**Table 4**). The 3- and 5-year local recurrence-free survival in patients with ID-type growth were 55.7% (95%CI, 30–81.4) and not reached, respectively, compared with 93.2% (95%CI, 84.1–100) and 93.2% (95%CI, 84.1–100) for those with pushing-type growth (p = 0.002, log rank test). There were no significant differences in the local recurrence-free survival between patients with ID- and IF-type growth or those with pushing- and IF-type growth. The 3- and 5-year local control rates in patients with IF-type growth were 71.3% (95%CI, 54.2–88.4) and 71.3% (95%CI, 54.2–88.4) (**Fig 3**).

**Table 4:** The prognostic factors for local control in 81 patients.

Variables		Univariate analysis			Multivariate analysis		
		HR	95% CI	p-value	HR	95% CI	p-value
Age	years	0.999	0.971–1.027	0.94			
Gender	Female	0.582	0.207–1.635	0.3			
	Male	1					
Grade	G2	0.608	0.199–1.855	0.38			
	G3	1					
Size	cm	1.163	1.081–1.25	<0.0001	1.156	1.073–1.244	0.0001
Depth	deep	3.879	0.515–29.194	0.19			
	s/c	1					
CRP	mg/dl	0.966	0.794–1.177	0.73			
NLR		1.155	0.864–1.545	0.33			
Surgical	positive	1					
margin	negative	0.52	0.169–1.601	0.25			
Growth	pushing	0.17	0.043–0.667	0.007	0.204	0.051–0.82	0.03
pattern	focal	0.484	0.171–1.367	0.17	0.687	0.231–1.244	0.5
	diffuse	1			1		

CRP; C-reactive protein, NLR; Neutrophil-lymphocyte ratio, s/c superficial, HR; Hazard ratio, 95%CI; 95% confidential interval

**Fig 4:**
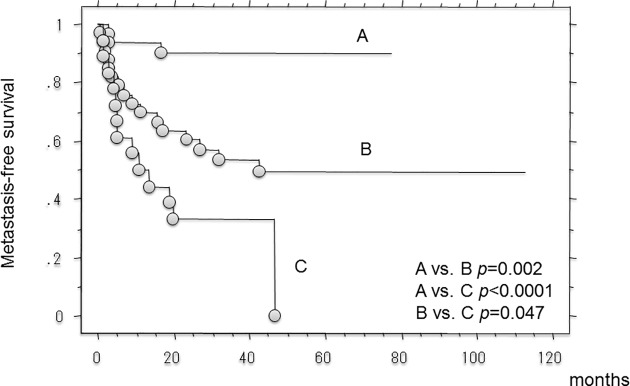
Kaplan-Meier curve showing the metastatic-free survival rates in the 81 patients with soft-tissue sarcomas. (A; patients with pushing-type infiltrative pattern, B; patients with focal-type infiltrative pattern, C; patients with diffuse-type infiltrative pattern).

Metastases were observed in 32 patients. The 3-year and 5-year metastasis-free survival rate was 61.6% (95%CI, 50.7–72.6) and 55.5% (95%CI,42.7–68.3). Univariate analysis revealed that growth pattern (pushing vs. ID: p = 0.0002and CRP (p <0.0001) were significantly associated with metastasis-free survival, and growth pattern (pushing vs. ID: p = 0.001) and CRP (p = 0.003) were independent prognosticators in the multivariate analysis (**Table 5**). The 3- and 5-year metastasis-free survival rates in patients with ID-type growth were 33.3% (95%CI, 11.6–55.1) and0%, respectively, compared with 89.9% (95%CI, 79–100) and 89.9% (79–100) for those with pushing-type growth (p < 0.0001, log rank test). There was a significant differences metastasis-free survival between patients with IF- and pushing-type growth (p = 0.002, log rank test). The 3- and 5-year metastasis-free survival rates in patients with IF-type growth were 53.7% (95%CI, 36.4–70.9) and 49.5% (95%CI, 31.8–67.3) (**Fig 4**). There was also a significant difference in metastasis-free survival among patients with focal- and ID-type growth (p = 0.03, log rank test).

**Table 5:** The prognostic factors for metastasis-free survival in 81 patients.

Variables		Univariate analysis			Multivariate analysis		
		HR	95% CI	p-value	HR	95% CI	p-value
Age	years	0.992	0.972–1.01	0.47			
Gender	Female	1.436	0.714–2.89	0.31			
	Male	1					
Grade	G2	0.597	0.258–1.38	0.23			
	G3	1					
Size	cm	1.027	0.969–1.08	0.37			
Depth	deep	1.387	0.533–3.60	0.5			
	s/c	1					
CRP	mg/dl	1.164	1.088–1.24	<0.0001	1.106	0.031–0.413	0.003
NLR		1.254	0.991–1.58	0.06			
Growth	pushing	0.089	0.025–0.31	0.0002	0.113	0.031–0.136	0.001
pattern	focal	0.488	0.231–1.03	0.06	0.576	0.262–1.201	0.17
	diffuse	1			1		

CRP; C-reactive protein, NLR; Neutrophil-lymphocyte ratio, s/c; superficial, HR; Hazard ratio, 95%CI; 95% confidential interval

## Discussion

In the present study, we demonstrated an association between the infiltrative growth pattern on MRI and elevations in the inflammatory markers, CRP and NLR. Tumor growth can cause tissue inflammation. Neutrophils (and sometimes eosinophils) are the effectors recruited first during the acute inflammatory response [13]. Monocytes, which differentiate into tissue macrophages, then migrate to the site of injury, guided by chemotactic factors. Once activated, tumor associated macrophages (TAMs) play a significant role in stimulating inflammatory infiltrates in tumor tissue. TAMs produce a number of potent angiogenic growth factor, cytokines and proteases, all of which are mediators that potentiate tumor progression [13]. The production of CRP in hepatocytes is induced principally at the transcriptional level following the elevation of circulating IL-6 [14]. Therefore, we consider that infiltrative growth increases the CRP levels and the NLR, although there was marginal significance of relationship between NLR and growth pattern. Elevated CRP and higher NLR have been associated with poor prognosis for survival in soft-tissue sarcoma [1–6].

We also found that univariate and multivariate analyses showed that the MRI growth pattern and elevated CRP were associated with disease-specific and metastasis-free survivals in patients with high-grade soft-tissue sarcoma. Furthermore, growth pattern was also associated with local tumor control. The infiltrative MRI growth patterns have been evaluated previously for UPS and MFS, with superficial tumors being more prone to demonstrate an infiltrative growth pattern.^7-12^ Conversely, Fernebro et al. reported that there were no differences in infiltrative growth patterns on MRI between the superficial and deep tumors [11]. Furthermore, their cohort consisted of patients with not only MFS and UPS but also other histologies such as LMS and LPS [11]. In the present study, 8 of 9 patients with superficial MFS and UPS showed focal-type infiltrative growth pattern. Additionally, MPNST and de-differentiated LPS were prone to demonstrate a ID-type growth pattern. There may be a difference of infiltration growth pattern between histological diagnosis. However, in present study, we could not show the difference of oncological outcome between IF and ID, although we divided infiltrative patterns into two types according to previous study [9]. Therefore, it may be important to evaluate whether tumor boarder is infiltrative or not.

In the present study, the local control rate in patients with a ID-type growth pattern was inferior to that in those with a pushing-type growth pattern. Several studies have demonstrated that an infiltrative growth pattern was significantly related to higher local recurrence rates [7,9,11,12]. Those studies included patients with low-grade MFS at the superficial layer. Low-grade MFS is unusual among low-grade sarcomas because it often recurs relentlessly and multiplies after surgical resection, despite gross negative margins and wide surgical resection [12,15]. On the other hand, Iwata et al. excluded patients with low-grade MFS, and reported that an infiltrative growth pattern was significantly correlated with poor disease-specific and metastasis-free survival rates, but was not associated with poor local control [10]. Therefore, the exclusion of low-grade sarcomas might account for the contrasting results to some previous studies. Furthermore, previous studies have demonstrated that tumor depth and histological grade were predictive factors for metastasis and survival, which could explain our findings that growth patterns were associated with inferior disease-specific and metastasis-free survivals [10,16–18]. It should be interesting whether adjuvant therapy, such as chemotherapy and radiotherapy, is effective for tumor control in patients with infiltrative growth pattern. Future randomized study could be expected.

This study has several limitations. First, histological examinations of the tumor periphery were not performed. In present study, there was no difference between surgical margin and infiltrative growth pattern. We usually evaluate surgical margin carefully. But we could not evaluate detail assessment of the site of infiltrative growth pattern on MRI. Inadequate assessment of surgical margins may have affected those results, especially for the patients with infiltrative pattern. Further histological evaluation should be necessary to validate our results, although there were discrepancies in the demarcation of the tumor area between MRI and histological examinations [9,19]. Second, this was a retrospective study with a small number of patients. Therefore, further prospective studies on a large-scale independent database are required to confirm the findings of the present study.

## Conclusions

There were significant associations between the tumor growth pattern and CRP levels in patients with high-grade soft-tissue sarcoma. An infiltrative growth pattern and elevated CRP were associated with inferior disease-specific and metastasis-free survival rates in these patients. Therefore, careful post-treatment follow-up should be conducted in such patients.
